# Comparison of Haseman-Elston regression analyses using single, summary, and longitudinal measures of systolic blood pressure

**DOI:** 10.1186/1471-2156-4-S1-S23

**Published:** 2003-12-31

**Authors:** Lucia Mirea, Shelley B Bull, James Stafford

**Affiliations:** 1 Samuel Lunenfeld Research Institute, Mount Sinai Hospital, 600 University Avenue, Toronto, Ontario, M5G 1X5, Canada; 2 Department of Public Health Sciences, University of Toronto, Toronto, Ontario, M5S 1A8, Canada

## Abstract

To compare different strategies for linkage analyses of longitudinal quantitative trait measures, we applied the "revisited" Haseman-Elston (RHE) regression model (the cross product of centered sib-pair trait values is regressed on expected identical-by-descent allele sharing) to cross-sectional, summary, and repeated measurements of systolic blood pressure (SBP) values in replicate 34, randomly selected from the Genetic Analysis Workshop 13 simulated data. RHE linkage scans were performed without knowledge of the generating model using the following phenotypes derived from untreated SBP measurements: the first, the last, the mean, the ratio of the change between the first and last over time, and the estimated linear regression slope coefficient. Estimates of allele sharing in sibling pairs were obtained from the complete genotype data of Cohorts 1 and 2, but linkage analyses were restricted to the five visits of Cohort 2 siblings. Evidence for linkage was suggestive (p < 0.001) at markers neighboring SBP genes Gb35, Gs10, and Gs12, but weaker signals (p < 0.01) were obtained at markers mapping close to Gb34 and Gs11. Linkage to baseline genes Gb34 and Gb35 was best detected using the first SBP measurement, whereas linkage to slope genes Gs10-12 was best detected using the last or mean SBP value. At markers on chromosomes 13 and 21 displaying strongest linkage signals, marginal RHE-type models including repeated SBP measures were fit to test for overall and time-dependent genetic effects. These analyses assumed independent sib pairs and employed generalized estimating equations (GEE) with a first-order autoregressive working correlation structure to adjust for serial correlation present among repeated observations from the same sibling pair.

## Background

Many complex quantitative traits (QT) such as blood pressure exhibit temporal variation and age-dependent penetrances, and as is the case with the Framingham Heart Study, are studied longitudinally. Statistical methods for QT linkage analyses include variance components (VC) analyses [1,2], traditional Haseman-Elston (HE) regression [3], "revisited" Haseman-Elston (RHE) regression [4], and other extensions to the RHE model [5-7]. Although multivariate trait analyses have been proposed for both RHE [4] and VC [8] methods, these methods are currently not available in commonly used software packages, and typically RHE and VC linkage analyses examine a single trait measurement for each subject. In the Framingham pedigrees, Levy et al. [9] performed a genome-wide VC linkage scan for systolic blood pressure (SBP), analyzing the residual obtained from regression of subject-specific mean SBP values vs. mean age and mean body mass index (BMI). de Andrade et al. [10] point out that this approach may be conservative and is unable to detect whether genetic variability is time dependent. They propose an extension to VC methods that can estimate genetic variability with serial observations and can test for temporal trends.

Predecessors of VC analyses, traditional HE models [3] regress the square of the difference between sib-pair trait values on the estimated proportion of marker alleles that the sib pair shares identically by descent (IBD). HE regression utilizes least-squares estimation and thus is simpler and more robust to non-normality than VC methods [11]. A series of extensions to the HE model employing a variety of transformations of sib-pair trait values can increase statistical power [4-7] and in some cases render HE analyses equivalent to VC techniques [12,13].

In this paper, we apply the RHE model to Genetic Analysis Workshop 13 (GAW13) simulated data to compare different strategies for conducting linkage analyses within the framework of a longitudinal study. The dependent variable in the RHE model is the cross product of sib-pair trait measures corrected for the sample mean. We also examine an RHE-type marginal model, similar to that of Ziegler et al. [14], that employs generalized estimating equations (GEE) [15] to accommodate the longitudinal measures from up to five exam visits and to facilitate a test for time-dependent genetic effects.

## Methods

### RHE genome scans of cross-sectional and summary SBP phenotypes

Replicate 34 was randomly selected from among the 100 simulated data sets available to GAW13, and single-point IBD sharing was estimated using the complete genotype data of Cohorts 1 and 2 via SAGE/GENIBD software [16]. Blind to the generating model, we performed RHE analyses with the program SAGE/SIBPAL [16] including only phenotypes from Cohort 2 siblings (2028 sibpairs) obtained at visits when subjects were not receiving treatment for hypertension. RHE analyses implemented in SAGE/SIBPAL utilize generalized least squares (GLS) to adjust for correlation between pairs of siblings from the same family. Separate genome scans examined the following four SBP phenotypes: first untreated measure (FirstSBP), last untreated measure (LastSBP), mean of all untreated measures (MeanSBP), ratio of change in SBP over time from first to last untreated measurement (ΔSBP) and the estimated linear regression slope coefficient (RegSBP). RHE models were fit to IBD sharing alone and then refit including sib-pair-level covariates (the mean-adjusted cross-product of covariate values for the sib pair). Covariates were selected that showed significant association with the SBP phenotype in prior individual-based univariate analyses. With knowledge of the generating model, we compared the ability of RHE analyses to detect linkage using the cross-sectional and summary phenotypes: FirstSBP, LastSBP, MeanSBP, ΔSBP, and RegSBP.

### Locus-specific RHE-type analyses with longitudinal SBP values

Based on the results of our genome scans and knowing the generating model, a marker on chromosome 13 and four markers on chromosome 21 were selected for longitudinal analyses. All these markers showed suggestive linkage evidence (*p *< 0.001) and were located close to SBP susceptibility genes. At these loci, the IBD-sharing estimates for Cohort 2 sib pairs were extracted from SAGE/GENIBD output, and using SAS software we calculated five different phenotypes: the mean-adjusted cross product of the FirstSBP, LastSBP, MeanSBP, ΔSBP, and RegSBP sib-pair values. RHE-type models that regressed these sib-pair cross products on IBD sharing were fit with SAS/GENMOD. Assuming no residual familial correlation among multiple sib pairs from the same pedigree, the RHE-type models were extended via GEE [15] to a marginal model including repeated sib-pair measurements.

GEE methods are a common statistical approach for analysis of generalized linear models with repeated measures in which a common correlation structure is specified for clusters of observations. A variance estimator is applied that is robust to misspecification of the correlation structure. In SAS/GENMOD, we fit a GEE longitudinal RHE-type model regressing mean-adjusted cross products of SBP values on IBD estimates. Clusters of repeated observations were defined at the sib-pair level assuming a first-order autoregressive (AR1) correlation structure. Specific to each visit, we considered the average age of the sib pair at the time of measurement adjusted for the overall population mean across measurements (SibAge). To test whether age had an influence on genetic variability, we included an interaction term between SibAge and IBD allele sharing. Similarly, we considered the visit number (1 to 5) and its interaction with IBD allele sharing to assess time-dependent effects.

## Results

### RHE genome scans of cross-sectional and summary SBP phenotypes

Linkage evidence provided by test results for significant IBD allele-sharing effects was comparable in models with and without covariates. Consistent with the description of Elston et al. [4], the inclusion of covariate main effects does not alter linkage tests but may help to explain additional variation in trait values not accounted for by a major gene. Therefore we present here only linkage results of IBD-allele-sharing effects in models without covariates. For markers mapping close to SBP slope genes Gs10-12, stronger evidence for linkage was observed using the LastSBP and MeanSBP measurements compared with the FirstSBP, ΔSBP, or RegSBP values. Significant evidence for linkage (*p *< 0.0001) was detected using MeanSBP at the two neighboring markers located on chromosome 21 within the interval between Gs12 and Gs10 (Table 1). Linkage to LastSBP and MeanSBP was suggestive (*p *< 0.001) at the other two markers, *GATA129D11 *and *GATA70B08*, on chromosome 21 flanking Gs12 and Gs10, respectively (Table 1). A weak linkage signal (*p *< 0.01) was seen at marker *GATA88H02 *on chromosome 15, mapping close to Gs11 (Table 1).

Among the cross-sectional and summary phenotypes examined, linkage to baseline genes Gb34 and Gb35 was best detected using the FirstSBP measurement. The evidence for linkage obtained using FirstSBP was suggestive (*p *< 0.001) at marker *ATA26D07 *on chromosome 13 close to Gb35 and weak (*p *< 0.01) at marker *GATA6E05 *on chromosome 5 close to Gb34 (Table 1). No linkage signal was observed on chromosome 7 close to Gb36.

### Locus-specific RHE-type analyses with longitudinal SBP values

In general, the RHE-type models fit to cross-sectional and summary phenotypes in SAS/GENMOD provide considerably less linkage evidence than analyses performed using SAGE/SIBPAL (Tables 1 and 2). An important difference between SAS/GENMOD and SAGE/SIBPAL is that estimation in SAS/GENMOD is equivalent to ordinary least squares (OLS) without adjustment for familial correlation, whereas SAGE/SIBPAL employs a GLS procedure that accounts for the correlation among related sibling pairs. SAGE/SIBPAL calculates residual correlation between cross-product values from sib pairs that share one or no common sibs using an estimate of the sibling trait correlation. The regression parameter estimates for IBD sharing obtained via GLS in SAGE/SIBPAL were larger and had smaller standard errors than those obtained via GEE in SAS/GENMOD. These differences may explain the observed discrepancies.

RHE-type analyses with repeated measures (Table 3) produced linkage results similar to corresponding models using MeanSBP values (Table 2). As a main effect, SibAge had a significant positive effect (Model 2 in Table 3), but the interaction between IBD sharing and SibAge was not significant (Model 3). Results were similar for analyses using visit number instead of SibAge. These negative findings suggest that nongenetic variability in SBP increases with age and time.

## Discussion

The structure of the simulated data mirrored that of the large complex Framingham pedigrees. IBD sharing among relative pairs was estimated using the full pedigrees, but to ease computations, linkage analyses examined only Cohort 2 sibling pairs. Based on the methods of Olson and Wijsman [17], extensions to RHE models have been proposed that consider other types of relative pairs [4], but release of the software SAGE/RELPAL is pending. In these analyses we have excluded observations when individuals were treated for hypertension, thus possibly eliminating measurements due to strong genetic effects and reducing the power to detect linkage.

As generated in these data, baseline genes Gb34-36 have a constant effect on SBP, but due to other time-dependent factors, the proportion of variation in SBP attributable to baseline genes decreases over time. It is therefore not surprising that linkage to Gb34-36 genes would be detected better using the FirstSBP measurement, which corresponds to younger ages. In contrast to baseline genes, the effect of slope genes Gs10-12 increases with age. Slope genes account for a greater proportion of SBP variability at older ages, and as expected, stronger linkage evidence is seen with the LastSBP and MeanSBP values than with the FirstSBP measurement. It is interesting that linkage evidence tended to be weaker for ΔSBP and RegSBP estimates than for LastSBP, MeanSBP, and longitudinal SBP values. As described in the GAW13 summary paper by Gauderman et al. [18], one plausible explanation is that large trait variability makes it difficult to detect gene effects that increase with time.

In a longitudinal approach, all data are utilized to assess simultaneously the presence of a genetic effect and test whether this effect is time-dependent. Thus a longitudinal model may be able to distinguish between baseline and slope genes. Such a longitudinal model must adjust for two levels of correlation: cross-sectional familial correlation between sib pairs from the same family and serial correlation among repeated measures on the same sib pair. GEE analyses in SAS/GENMOD are limited to one level of clustering, which we used to correct for serial correlation while ignoring familial correlation. The GEE is a marginal or population-average model, so the RHE-type models fit in SAS/GENMOD using GEE effectively pool sib-pair observations across multiple visits. Because the sib-pair IBD sharing does not depend on age or time, it is a constant cluster covariate and the phenotype-genotype association is averaged across multiple visits. Inclusion of an interaction term between IBD sharing and SibAge or visit number allows for the possibility that, for example, the sib-pair phenotype-genotype association is higher in younger sib pairs.

The GEE method provides a robust variance estimate but may be less efficient than other longitudinal models that make stronger assumptions. Methods more efficient than GEE could be used to extend RHE-type models to include repeated measurements and adjust for both familial and serial correlation. For example, cross-sectional and longitudinal correlation could be jointly parameterized in a GLS approach. Another possibility is to apply a mixed effects model that accommodates cross-sectional and sib-pair-specific effects.

## Conclusions

The RHE scan for cross-sectional and summary SBP phenotypes identified five of six SBP susceptibility genes (using a criterion of *p *< 0.01), and thus this approach remains an important tool for QT linkage analyses. A more stringent testing level of *p *< 0.001 for MeanSBP was sufficient to eliminate the three apparent false positive results at the price of two false negatives (Gb34, Gs11). Analyses using early phenotypes may be more powerful to detect baseline genes whose contribution to trait variance decreases over time. However, analyses using single phenotype measures are unable to distinguish between genes with a constant effect and those with a variable age effect. As seen in these data, when the gene effect increases with age, a longitudinal model may be preferable because it provides comparable results to mean summary values, but facilitates a test for interaction between age/time and gene effects.

## Figures and Tables

**Table 1:** Single and summary measures RHE linkage *p*-values (one-sided) for IBD allele sharing (SAGE/SIBPAL)^A^

**Chr**	**Gene (cM)^B^**	**Marker (cM)^B^**	**First SBP**	**Last SBP**	**Mean SBP**	**ΔSBP**	**Reg SBP**
**True positive results (*p *< 0.001) at markers linked to SBP genes**							

**13**	Gb35 (85.2)	***ATA26D07 ***(90.8)	0.0002	0.04	0.001	0.88	0.94
**21**	Gs12 (29.5)	***GATA129D11 ***(26.6)	0.02	0.0008	0.004	0.11	0.0009
		***ATA27F01 ***(40.0)	0.0005	0.0002	0.00005	0.003	0.006
	Gs10 (53.6)	***GATA188F04 ***(44.9)	0.001	0.00001	0.00001	0.005	0.0007
		***GATA70B08 ***(63.4)	0.06	0.0002	0.002	0.002	0.001

**False negative results (*p *> 0.001) at markers near SBP genes**							

**5**	Gb34 (176.1)	***GATA6E05 ***(174.6)	0.008	0.28	0.09	0.42	0.21
**7**	Gb36 (47.5)	**GGAA3F06 **(45.2)	0.43	0.47	0.44	0.32	0.39
		**GATA13G11 **(54.6)	0.47	0.84	0.74	0.27	0.51
**15**	Gs11 (4.5)	***GATA88H02 ***(13.2)	0.01	0.01	0.009	0.50	0.034

**False positives (*p *< 0.001) at markers with no linkage to SBP genes**							

**2**	-	***GATA4G12 ***(237.5)	0.0007	0.004	0.003	0.04	0.10
**9**	-	***GATA21A06 ***(24.2)	0.0008	0.15	0.01	0.95	0.84
**17**	-	***ATA43A10 ***(96.4)	0.80	0.045	0.31	0.02	0.0008

^A^RHE models (SAGE/SIBPAL) employed GLS methods with adjustment for familiar correlation. ^B^Positions (cM) correspond to Haldane sex-average map.

**Table 2:** Single and summary measures RHE linkage *p*-values (one-sided) for IBD allele sharing (SAS/GENMOD)^A^

**Chr**	**Gene (cM)^B^**	**Marker (cM)^B^**	**First SBP**	**Last SBP**	**Mean SBP**	**ΔSBP**	**Reg SBP**
**13**	Gb35 (85.2)	***ATA26D07 ***(90.8)	0.0003	0.06	0.003	0.14	0.09
**21**	Gs12 (29.5)	***GATA129D11 ***(26.6)	0.10	0.08	0.08	0.31	0.04
		***ATA27F01 ***(40.0)	0.05	0.07	0.04	0.04	0.03
	Gs10 (53.6)	***GATA188F04 ***(44.9)	0.09	0.004	0.002	0.06	0.02
		***GATA70B08 ***(63.4)	0.09	0.01	0.03	0.20	0.09

^A^RHE models (SAS/GENMOD) employed OLS methods without adjustment for familial correlation. ^B^Positions (cM) correspond to Haldane sex-average map.

**Table 3:** Longitudinal measures RHE linkage *p*-values, (one-sided) for IBD allele sharing and (two-sided) for SibAge (SAS/GENMOD)

			**Model 1**	**Model 2**		**Model 3**		
					
**Chr**	**Gene (cM)^A^**	**Marker (cM)^B^**	IBD	IBD	SibAge	IBD	SibAge	IBD* SibAge
**13**	Gb35 (85.2)	***ATA26D07 ***(90.8)	0.006	0.006	0.00001	0.007	0.005	0.78
**21**	Gs12 (29.5)	***GATA129D11 ***(26.6)	0.09	0.08	0.00001	0.09	0.0007	0.80
		***ATA27F01 ***(40.0)	0.04	0.03	0.00001	0.04	0.006	0.92
	Gs10 (53.6)	***GATA188F04 ***(44.9)	0.003	0.003	0.00001	0.003	0.001	0.91
		***GATA70B08 ***(63.4)	0.09	0.08	0.00001	0.10	0.002	0.64

^A^Longitudinal RHE models (SAS/GENMOD) employed GEE methods with an AR1 working correlation structure to adjust for serial correlation, but without adjustment for familial correlation. ^B^Positions (cM) correspond to Haldane sex-average map.
